# 

**Published:** 1865-12

**Authors:** 


### Editorial.

### CLOSE OF THE VOLUME.

The present number concludes the Nineteenth Volume of the REGISTER ; with it we send bills to all who are in arrears upon the subscription book, and we hope that a very early response will be made to them all. And if we should by mistake or oversight fail to send bills to any who are entitled to them, we are willing to be forgiven, and will make immediate reparation upon being notified of the fact. There are upon our subscription book a few names which we have been writing upon the wrappers of the REGISTER for the last four years, without obtaining one particle of acknowledgment of the fact in any way whatever. Well, this may do for four or five years, but if it should continue for ten or twelve it would become a nuisance. We do not want to agitate anybody; but really it would be pleasant to receive occasionally a word of encouragement if nothing more, or at least some indication by which we may know what your feelings are, if you are pleased say so, if you are disgusted say so, and then we will know whether to waste any more paper and printer's ink upon you or not.

### BACK NUMBERS.

We are often called upon to furnish back numbers of the REGISTER, this we are pleased to do when the number desired is not exhausted. In almost every volume some of the numbers become exhausted before the close of the year. It is impossible to keep a large number of full files. We hope persons will not feel disappointed when we cannot furnish numbers they may call for. All who value the REGISTER should be careful to preserve them. We have quite a quantity of miscellaneous numbers which will be distributed as occasion may require.

### THE TWENTIETH VOLUME.

With the January number begins the Twentieth Volume of the "DENTAL REGISTER," the oldest dental journal in existence, so far as we are aware.

What it shall be will depend more upon the profession than on the editor. If it receives the proper aid from the *pens* and *pockets* of the profession, it can be made of inestimable value.

We desire very much to have contributions from the pens of all who can write; we do not ask or wish for the exclusive use of the writings of any, but we do hope to receive a proper share from all. We do not propose to pay for contributions as a rule, we hold out no such grand inducements. It should not be expected, and will not be expected or desired by those who have at heart the best interests of the profession.

The REGISTER is self-sustaining, and must be; but, however prosperous and successful it may become all the proceeds will be expended upon it as heretofore. Our labor upon the REGISTER, which is not small or light, has been pecuniarily a free contribution to the profession, and will probably always be so.

For the kind regard which is so largely entertained in the profession, for the REGISTER, we are very thankful, indeed, and in that find a large reward for our labor. The REGISTER has arisen to that point when we think some change is practicable and indeed demanded. The first change proposed, is in the title, viz : to drop that part which has hitherto designated it a western institution, which was proper enough in the beginning, but is now objectionable — It can't afford to be confined to the west. It will henceforth be "THE DENTAL REGISTER."

Several other changes are proposed, which will be apparent when it is issued.

It will be continued the same size as heretofore, though heavier paper will be used.

It will be very pleasant and desirable to receive early subscriptions upon this volume, that it may not start out in debt.

### TESTIMONIALS.

This is to certify that I have used the various and most of the different manufacturers teeth for the last eight years, and now give to Johnson & Lund's the *choice;* deeming them as combining the most desirable qualities looked for in artificial teeth; such as NATURALNESS OF COLOR, SHAPE, SYMMETRY and BEAUTY OF ARRANGEMENT, COMELINESS OF EXPRESSION in the mouth, and STRENGTH and FIRMNESS for use.

R. WALKER, Oswego, New York.

---

MESSRS. JOHNSON & LUND:

*Gents*—I have had an opportunity of using some of your beautiful teeth, and can pronounce them *Excelsior.* They stand the fire well, and are in every respect worthy.

S. J. BRACKETT, St. Louis, Mo.

---

Having within the last thirteen years used teeth from nearly every establishment in the Union, I have no hesitation in saying that Johnson & Lund's Teeth, as lately improved, present a greater combination of desirable qualities than do those of any other one establishment; and I cheerfully recommend them to the profession *as a decidedly superior article.*

F. O. HYATT, Cortlandville, N. Y.

---

If there is such a thing as *perfection* in the manufacture of artificial teeth, Johnson & Lund have certainly attained it.

E. J. LARASON, Philadelphia.

---

MESSRS. JOHNSON & LUND:

*Gentlemen*—Having used your teeth in my practice for the past year, and finding them SUPERIOR to all others in BEAUTY, NATURAL APPEARANCE, and DURABILITY, I hereby add my humble testimony in their favor.

Yours, respectfully, B. F. CLARK, Flint, Michigan.

---

I have used teeth from the different manufacturers for ten years, and have discovered in none those qualities which so nearly approach a desideratum as the productions of Johnson & Lund. Their resemblance to nature in shape. shade and transparency is unsurpassed; and their freedom from brittleness under the hammer, and unequalled endurance under the blow-pipe, show that the proportion of their ingredients effect such a happy combination as to leave little room for improvement in respect to general strength.

S. H. COSTEN, Philadelphia

---

I use Johnson & Lund's make of Artificial Teeth, and can recommend them as being perfect in every particular; they give *complete satisfaction,* and leave nothing further to be desired.

J. M. BARRETT, Wilkesbarre, Pa

---

MESSRS. JOHNSON & LUND:

*Dear Sirs*—I take pleasure in adding my certificate in favor of your teeth. *They are without a fault.*

A. H. FOWLER, Ithica, New York.

---

MESSRS. JOHNSON & LUND:

*Gentlemen*—You ask me how I like your teeth. I must say that I prefer them to those of any other manufacturer. The *beautiful shape,* the *life-like* and *natural* shades, and their *strength* under the *blow-pipe* and *hammer,* render them all that the dentist could desire. The perfection you have attained in the manufacture of artificial teeth, deserves the thanks and substantial support of the dental profession.

Wishing you success, I remain. yours truly,

M. LUKENS LONG, Philadelphia.

---

Having used Johnson & Lund's Teeth to my perfect satisfaction, I would earnestly recommend them to Dentists in search of a superior article, and one that will *always please.*

S. F. TREMAIN, (of the firm of Tremain & Bro's.) Rome. N. Y.

---

MESSRS. JOHNSON & LUND:

Having during the past year used five hundred dollars worth of your teeth, we have come to the conclusion to use none other now manufactured.

HOLBROOK, BUTLER & HUNTINGTON.

Watertown, N. Y., March 23, 1863.

### PENNSYLVALIA COLLEGE OF DENTAL SURGERY.

### THE TENTH ANNUAL SESSION, 1865–66.

### FACULTY.

J. D. WHITE, D.D.S., Emeritus Professor.

T. L. BUCKINGHAM, D.D.S., Prof. of Chemistry and Metallurgy.
E. WILDMAN, D.D.S., Prof. of Mechanical Dentistry.
G. T. BARKER, D.D.S., Prof. of Principles of Dental Surgery and Therapeutics.
W. S. FORBES, D.D.S., Prof. of Anatomy and Physiology.
JAMES TRUMAN, D.D.S., Prof. of Dental Physiology and Operative Dentistry.
EDWIN T. DARBY, D.D.S., Demonstrator of Operative Dentistry.
J. M. BARSTOW, D.D.S., Demonstrator of Mechanical Dentistry.

The regular Course will commence on the first Monday of November, and continue until the first of March ensuing. During October the Laboratory will be open, and a Clinical Lecture delivered every Saturday, by one of the Professors, at 3 o'clock, P. M. The most ample facilities are furnished for a thorough course of practical instruction.

| | |
|---|---|
| Tickets for the Course, Demonstrators' Tickets included, | $100 |
| Matriculation Fee, - - - - - - - - | 5 |
| Diploma Fee, - - - - - - - - | 30 |

For further information address

T. L. BUCKINGHAM, Dean,
243 North Ninth Street, Philadelphia.

### OHIO COLLEGE

OF

### DENTAL SURGERY.

The Twentieth Annual Session of the Ohio College of Dental Surgery will commence on the first Monday of November, and continue to the 20th of February succeeding.

### FACULTY.

JAMEL TAYLOR, M. D , D. D. S.,
Emeritus Professor of Institutes of Dental Science.

C. W. SAALDING, M. D., D. D. S.,
Professor of Institutes of Dental Science.

C. KEARNS, M. D.,
Professor of Anatomy and Physiology.

H. R. SMITH, M. D , D. D. S.,
Professor of Mechanical Dentistry and Metallurgy.

GEORGE WATT, M. D., D. D. S.,
Professor of Dental Chemistry and Therapeutics,

J. TAFT, D. D. S.,
Professor of Operative Dentistry and Dental Hygiene.

L. D. WALTER, D. D S., (Late of Rochester, N. Y.)
Demonstrator of Operative Dentistry.

J. G. VAN MARTER, D. D. S.,
Demonstrator of Mechanical Dentistry.

WILL TAFT, D. D. S.
Demonstrator of Anatomy.

### TEXT BOOKS.

Gray's and Wilson's Anatomy; Dalton's Physiology; Williams' Pathology; Fown's, Turner's, Graham's, or Brandt and Taylor's Chemistry; Taft's Operative Dentistry; Richardson's Mechanical Dentistry; Harris' Principles and Practice of Dental Science.

### TERMS OF ADMISSION.

Ticketr must be procured at the beginning of the session.

| | |
|---|---|
| Tickets for the course, | $100 00 |
| Demonstrator's Ticket for Anatomy, | 5 00 |
| Matriculation Fee, but once, | 5 00 |
| Diploma Fee, | 30 00 |

### TERMS OF GRADUATION.

Two courses, (including one of dissections,) with a dental pupilage.
Four years reputable practice will be received as equal to one course.
An acceptable thesis upon some subject on Dental science.
Must possess a good moral character, and be twenty-one years old.

For further information, address J. TAFT, *Dean.*
172 Race St.

### BALTIMORE COLLEGE OF DENTAL SURGERY.

TWENTY-SIXTH ANNUAL SESSION, 1865–66.

### FACULTY.

THOMAS E. BOND, A.M., M.D., Prof. of Pathology and Therapeutics.
PHILIP H. AUSTEN, A.M., M.D., D.D.S., Prof. of Dental Science and Mechanism and of Physiology.
FERDINAND J. S. GORGAS, A.M., M.D., D.D.S., Prof, of Dental Surgery and of Anatomy.
WILLIAM E. AIKEN, M.M., L.L.D., Prof. of Chemistry.
HENRY HOBART KEECH, D.D.S., Demonstrator of Operative Dentistry.
THOMAS SOLLERS WATERS, D.D.S., Demonstrator of Mechanical Dentistry.

The *Twenty-Sixth* Annual Session will commence on the first of November, 1865, and close on the first of March, 1866.

| | |
|---|---|
| Lecture and Demonstration Fees, - - - | $115 |
| Matriculation Fee (paid only once) - - - - | 5 |
| Diploma Fee. - - - - - - - | 30 |

For information, address

F. J. S. GORGAS, M.D., Dean of the Faculty.
*No. 43 Hanover St. Baltimore, Md.*

### PHILADELPHIA DENTAL COLLEGE

108 North Tenth St., above Arch.

THIRD ANNUAL SESSION, 1865-66.

### TRUSTEES.

PRESIDENT.

REV. RICHARD NEWTON, D.D.

SECRETARY.

R. SHELTON MACKENZIE, D.C.L.

PETER F. ROTHERMEL,

WILLIAM DULTY,

GEORGE J. ZIEGLER, M.D.,

GEORGE WILLIAMS,

ROBERT L. McCLELLAN, D.D.S.

J. L. SUESSEROTT, M.D., D.D.S..

COLSON HIESKELL,

S. FISHER CORLIES,

JAMES L. CLAGHORN,

EX-GOV. JAS. POLLOCK,

CHARLES S. BECK, M.D.

HENRY CRUMLEY.

### FACULTY

C. A. KINGSBURY, D.D.S.,
Emeritus Professor of Dental Physiology and Operative Dentistry.

HENRY MORTON, A.M.,
Emeritus Professor of Chemistry.

GEO. W. ELLIS, D.D.S.,
Professor of Dental Physiology and Operative Dentistry

THOS. WARDLE, D.D.S.,
Professor of Mechanical Dentistry and Metallurgy.

J. H. McQUILLEN, D.D.S.,
Professor of Anatomy, Physiology and Hygiene.

J. FOSTER FLAGG, D.D.S.,
Professor of Institutes of Dentistry.

ALBERT R. LEEDS, A. M..
Professor of Chemistry.

T. C. STELLWAGEN, D.D.S.,
Demonstrator of Operative Dentistry

WM. P. HENRY, D.D.S.,
Demonstrator of Mechanical Dentistry.

The Dispensary and Laboratory of the College will be open, and preliminary lectures will be delivered by one of the Professors every day during the latter half of October; commencing on the 17th instant, the lecture on Wednesday of each week, at 3 o'clock P.M., to be devoted to *Clinical* teaching. The regular Course of instruction will commence on the first Monday of November, and continue until the close of the ensuing February.

The lectures will be amply illustrated by the extensive and *valuable* collections of Anatomical, Pathological, and Mineralogical specimens, and the Philosophical and Chemical apparatus of the incumbents of the various Chairs, and every opportunity will be afforded in the Clinic and Laboratory for obtaining a *practical* knowledge of Operative and Mechanical Dentistry.

### FEES.

| | |
|---|---|
| Matriculation (paid but once) - - - - - - | $5 00 |
| Tickets for the Course, including the Demonstrators' - | 100 00 |
| Diploma - - - - - - - - - - | 30 00 |

For further particulars, address J. H. McQUILLEN, DEAN,
1112 *Arch Street, Philadelphia.*

y '65.

### Railroad Time Table.

Trains run as follows, Sundays excepted:

LITTLE MIAMI.

| | Departs. | Arrives. |
|---|---|---|
| Cincinnati Express | 6.00 A. M. | 7.30 P. M. |
| Mail | 9 00 A. M. | 4.31 A. M. |
| Columbus Accommodation, | 4.00 P. M. | 11.30 A. M. |
| Morrow Accommodation | 5.20 P. M. | 8.00 A. M. |
| Night Express, | 10.00 P. M. | 10.05 A. M. |

MARIETTA & CINCINNATI.

| | | |
|---|---|---|
| Mail | 8.00 A. M. | 7.00 P. M. |
| Accommodation | 3.30 P. M. | 11.00 A. M. |

CINCINNATI, HAMILTON & DAYTON

| | | |
|---|---|---|
| Indianapolis and Cambridge City | 6.00 A. M. | 5.50 P. M. |
| Toledo and Detroit | 7.00 A. M. | 9.50 P. M. |
| Dayton and Sandusky | 7.00 A. M. | 5.50 P. M. |
| Richmond and Chicago | 7.00 A. M. | 9.50 P. M. |
| Dayton Accommodation, | 2.10 P. M. | 9.50 P. M. |
| Indianapolis and Cambridge City | 4.00 P. M. | 12.25 P. M. |
| Toledo, Detroit and Huntsville | 5.00 P. M. | 12.50 P. M. |
| Hamilton Accommodation | 7.00 P. M. | 6.45 A. M. |
| Richmond and Chicago | 6.00 P. M. | 12.25 P. M. |
| Hamilton Accommodation | ......... | 8.00 A. M. |

INDIANAPOLIS & CINCINNATI.

| | | |
|---|---|---|
| Chicago and St. Louis Express | 7.00 A. M. | 10.00 A. M. |
| Springfield and St. Joseph Express | 1.00 P. M. | 5.07 P. M. |
| St. Louis and Chicago Express | 5.00 P. M. | 2.00 A. M. |
| Lawrenceburg and Harrison Accom | 5.15 P. M. | 8.10 A. M. |
| Harrison Accommodation | 9.30 A. M. | 2.40 P. M. |

CINCINNATI & INDIANAPOLIS JUNCTION.

| | | |
|---|---|---|
| onnersville, Cambridge City and Indianapolis Mail | 7.00 A. M. | 9.45 P. M. |
| Connersville, Cambridge City and Indianapolis Express | 3.30 P. M. | 12.50 P. M. |

OHIO & MISSISSIPPI.

| | | |
|---|---|---|
| St. Louis, Cairo and Louisville | 7.20 A. M. | 8.00 A. M. |
| St Louis Cairo and Louisville, | 7.50 P. M. | 11.00 P. M. |
| Louisville, Special Train | 3.45 P. M. | 1.50 P. M. |
| One train on Sunday through to St. Louis. | 7.50 P. M. | |

CINCINNATI, & ZANESVILLE.

| | | |
|---|---|---|
| Mail | 9.00 A. M. | 7.30 P. M. |
| Caboose | 4.00 P. M. | 8.00 A. M. |

COVINGTON & LEXINGTON.

| | | |
|---|---|---|
| Express, | 6.00 A. M. | 6.00 P. M |
| Accommodation | 2.35 P. M. | 10.12 A. M |

ATLANTIC & GREAT WESTERN RAILWAY.

| | | |
|---|---|---|
| Lightning Express | 6.00 A. M. | 6,30 P. M |
| Express Mail | 9.40 A. M. | 4.30 A. M. |
| Night Express | 10.50 P. M. | 7.30 P. M |

### CONTENTS.

### The Dental Register

### OF THE WEST.

*Issued Monthly, at $3 a Year in Advance.*

DEVOTED TO THE INTERESTS OF THE DENTAL PROFESSION.

**EDITED BY J. TAFT**

The volume commences in January and closes in December.

The Register being issued monthly is always fresh, therefore indispensable to the practitioner who desires to keep posted up with the new inventions and improvements which are daily developed. Neither expense nor effort will be spared to give value and variety to its contents. Articles will be illustrated with well executed engravings whenever they will add to the interest or assist in explanation.

Its extensive circulation and frequency of issue makes it one of the BEST DENTAL ADVERTISING MEDIUMS in the United States.

RATES OF ADVERTISING.

One page, 1 year, $50. Half page, 1 year, $30. Quarter page, or card, 1 year $20.

All advertising bills payable in advance, or at furthest after the issue of the first number containing the advertisement. All transient advertisements to be paid for in advance.

☞ CONTRIBUTIONS SOLICITED.

Address, J. TAFT, or "DENTAL REGISTER," Cincinnati, Ohio.

Business Communications to

**J. TAFT, Publisher,**
No. 172 Race Street.

